# Development of ricehusk ash reinforced bismaleimide toughened epoxy nanocomposites

**DOI:** 10.3389/fchem.2014.00065

**Published:** 2014-09-16

**Authors:** K. Kanimozhi, K. Sethuraman, V. Selvaraj, M. Alagar

**Affiliations:** ^1^Department of Chemical Engineering, Alagappa College of Technology, Anna UniversityChennai, India; ^2^Department of Chemistry, University College of Engineering Villupuram, (A Constituent College of Anna University, Chennai)Villupuram, India

**Keywords:** epoxy, bismaleimide, rice husk ash, 3-glycidoxypropoyltrimethoxysilane (GPTMS), AFM, TEM, mechanical and thermal properties

## Abstract

Recent past decades have witnessed remarkable advances in composites with potential applications in biomedical devices, aerospace, textiles, civil engineering, energy, electronic engineering, and household products. Thermoset polymer composites have further enhanced and broadened the area of applications of composites. In the present work epoxy-BMI toughened-silica hybrid (RHA/DGEBA-BMI) was prepared using bismaleimide as toughener, bisphenol-A as matrix and a silica precursor derived from rice husk ash as reinforcement with glycidoxypropyltrimethoxysilane as coupling agent. Differential scanning calorimetry, electron microscopy, thermogravimetric analysis, and goniometry were used to characterize RHA/DGEBA-BMI composites developed in the present work. Tensile, impact and flexural strength, tensile and flexural modulus, hardness, dielectric properties were also studied and discussed. The hybrid nanocomposites possess the higher values of the glass transition temperature (Tg) and mechanical properties than those of neat epoxy matrix.

## Introduction

The present work involves the development of an organic and inorganic hybrid in combination with a recycling waste material (rice husk ash) to enhance the properties of organic polymer. The wide spread uses and properties of epoxy resin have been extensively studied by our research group for the past two decades (Dinakaran et al., 2003; Rajasekaran and Alagar, 2007; Premkumar et al., 2008; Ramesh et al., 2010; Selvaganapathi et al., 2010; Chandramohan and Alagar, 2011; Vengatesan et al., 2011; Kanimozhi et al., 2013; Prabunathan et al., 2013). In the present study an attempt has been made to form a covalent bond between organic polymers and inorganic components through coupling agent to enhance the compatibility of components involved (Farhadyar et al., 2005; Bagherzadeh and Mahdavi, 2007; Xinghong et al., 2007; Qingming et al., 2008; Balamurugan and Kannan, 2010; Sea-Fue et al., 2010).

Bismaleimide resins (BMI) are thermosetting polyimides that can be polymerized via multiple carbon–carbon bond formations without generating volatiles. The thermal curing properties of BMI and their incorporation with epoxy matrices lead to possess superior thermal and flame-retardant properties in comparison with those of conventional epoxy resins. BMI-modified epoxy resin matrices have been shown to have high crosslinking ability and glass transition temperature, high thermal stability and char yield, excellent fire resistance, specific strength and specific modulus and lower water absorption (Gu et al., 1996; Chanda and Rahabi, 1997; Dinakaran et al., 2003; Dinakaran and Alagar, 2004; Qilang et al., 2010). Rice husk ash possesses hard surface, high silica content (80–90%), insoluble in water, possess high chemical stability and mechanical strength, abrasive in nature, inherent resistance behavior, small bulk density, non-toxicity, low cost, and stable to bacteria (Krishnarao et al., 1998; Hanafi et al., 2001; Qiang et al., 2008; Li et al., 2011; Yue et al., 2011). Due to the high content of silica moiety in the rice husk ash it is expected to function similar to OMMT-clay with epoxy polymer and hence it has been chosen as the inorganic reinforcement in the present work. (Khalf and Ward, 2009; Kumagai and Sasaki, 2009; Bhagiyalakshmi et al., 2010; Adam et al., 2012; Chand et al., 2012). The present investigation was focused on the development of new epoxy based hybrid matrices reinforced chemically with functionalized rice husk ash and toughened by N,N′ bismaleimido 4,4′ diaminodiphenylmethane cured using DDM. The resulting hybrid organic-inorganic composites were characterized by different analytical methods and the data resulted are reported and discussed.

## Experimental and materials

GPTMS, ethanol, diaminodiphenylmethane (DDM), were obtained from SRL (India) and were used as received. The matrix resin used in the present study was diglycidyl ethers of bisphenol—A (DGEBA epoxy resin) (LY556) was received from Ciba-Geigy Ltd. Rice husk ash was synthesized as per the reported procedure (Bhagiyalakshmi et al., 2010).

### Preparation of rice husk ash (RHA)

Acid treatment is one of the most useful routes used to remove the lignin, wax and oils covering the external surface of the fiber cell wall of natural fibers. Rice husk ash is a solid obtained after burning of rice husk and was washed with distilled water, dried in an oven at about 60°C for 2 h. Then bleached with conc. HCl to remove the dirt and other contaminants present in it and subsequently washed with water till the pH become neutral, then dried in oven at 60°C for 4 h. Then it was heated at 500°C for 5 h in a muffle furnace, to obtain rice husk ash.

### Surface functionalization of rice husk ash (GRHA)

**Figure 1:**
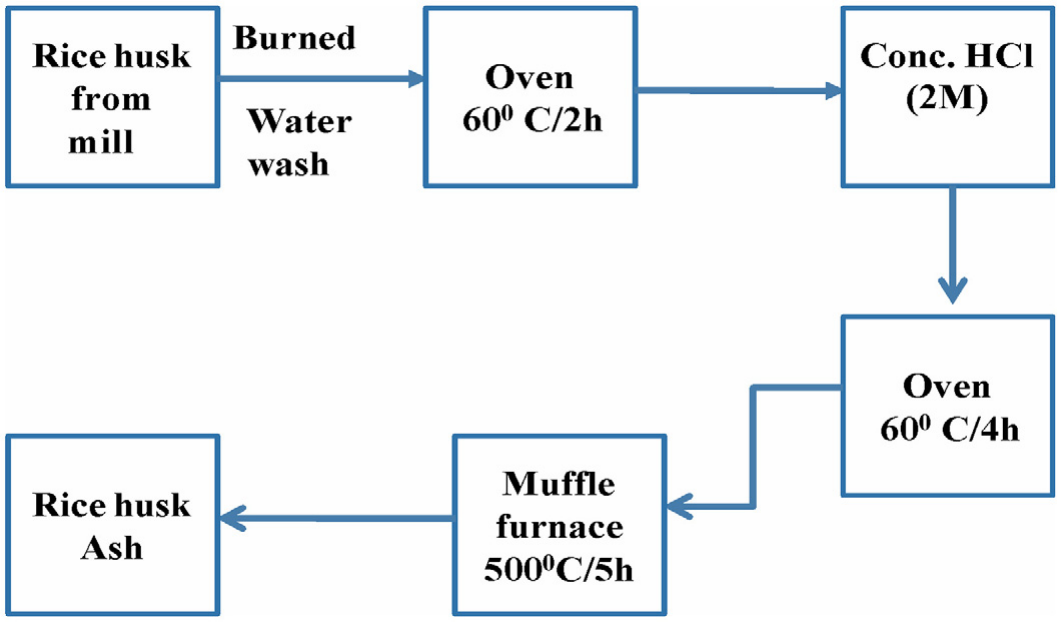
**Synthesis of rice husk ash (RHA) from rice hull**.

3-glycidoxypropyltrimethoxysilane (GPTMS) was used as coupling agent to functionalize the rice husk ash. Four milliliter ml of GPTMS was mixed with 95% absolute ethanol and 5% deionized water and the resulting solution was sonicated for 15 min. The pH of the solvent was initially adjusted to 4.5 using acetic acid and subsequently sonicated for 1 h in order to get complete hydrolysis of GPTMS. Then 10 g of rice husk ash was added and the resulting mixture was sonicated for 2 h. Then the mixture was refluxed for 24 h at 80°C and centrifuged with addition of water followed by ethanol and hexane. The rice husk ash thus functionalized was further dried in hot air oven at 100°C in order to remove the moisture (Figure 1).

### Synthesis of N,N′ bismaleimido 4,4′ diaminodiphenyl methane (BMI)

To a 1-l three-necked round-bottom flask fitted with paddle stirrer, reflux condenser and nitrogen inlet, were added, 600 ml acetone, 1.0 mole (98.1 g) maleic anhydride and 0.5 mole of the diaminodiphenylmethane. Rapid formation of precipitate of the bismaleiamic acid occurred on mixing the reactants together, and the mixture was allowed to stand for 30 min to complete the reaction (Ashok kumar et al., 2002). To the above, 1.0 g of nickel acetate and 25 ml of triethylamine were added and the entire mixture was heated slowly to reflux. Then by means of pressure equalizing funnel 117.9 ml acetic anhydride was added to the refluxing reaction mixture and heating was continued for an additional 3 h. The reaction mixture was diluted with 500 ml water and chilled to crystallize the bismaleimide (Yield 92%).

### Preparation of RHA/DGEBA-BMI nanocomposites

**Scheme 1:**
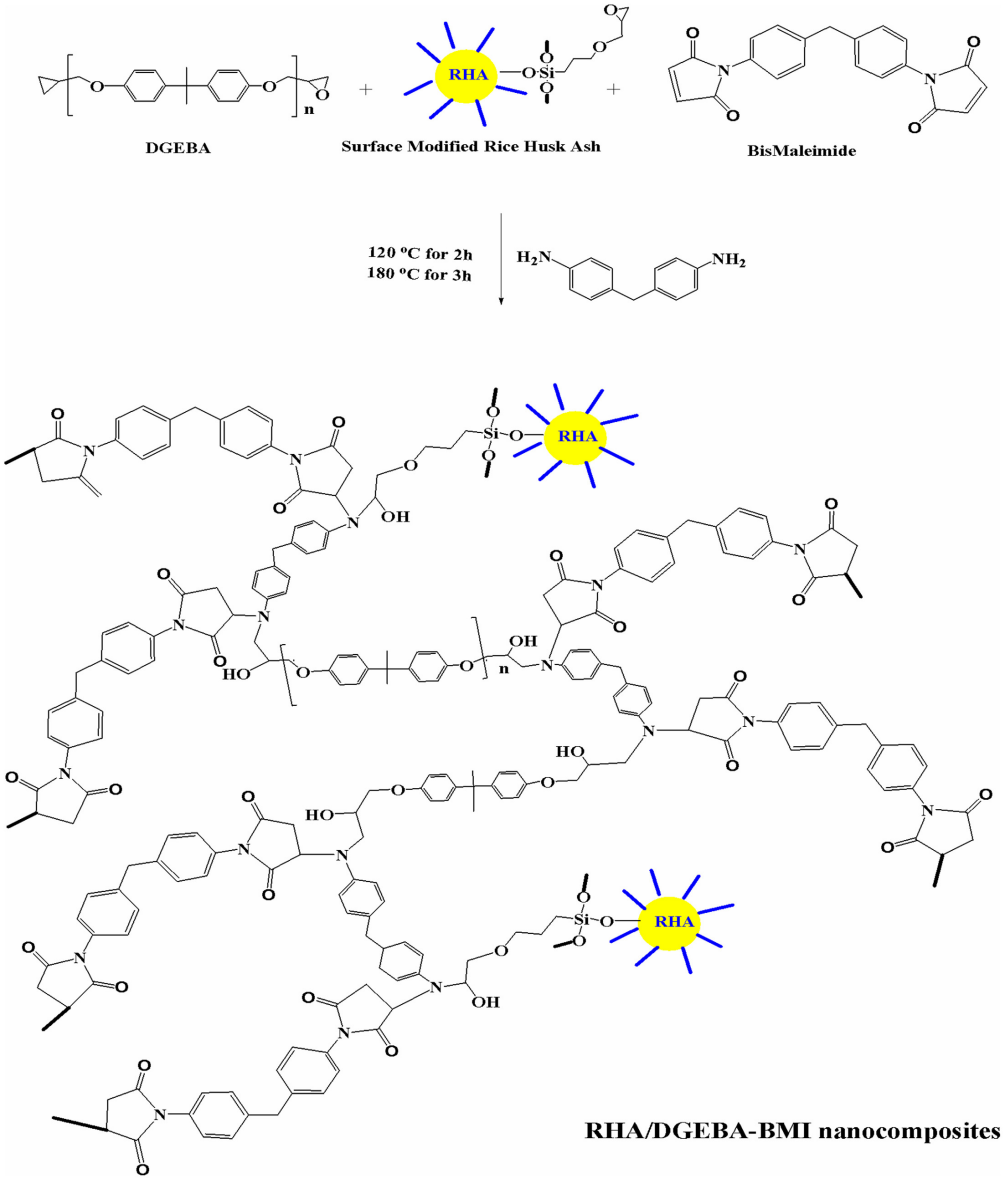
**Schematic representation of RHA/DGEBA-BMI nanocomposites**.

**Figure 2:**
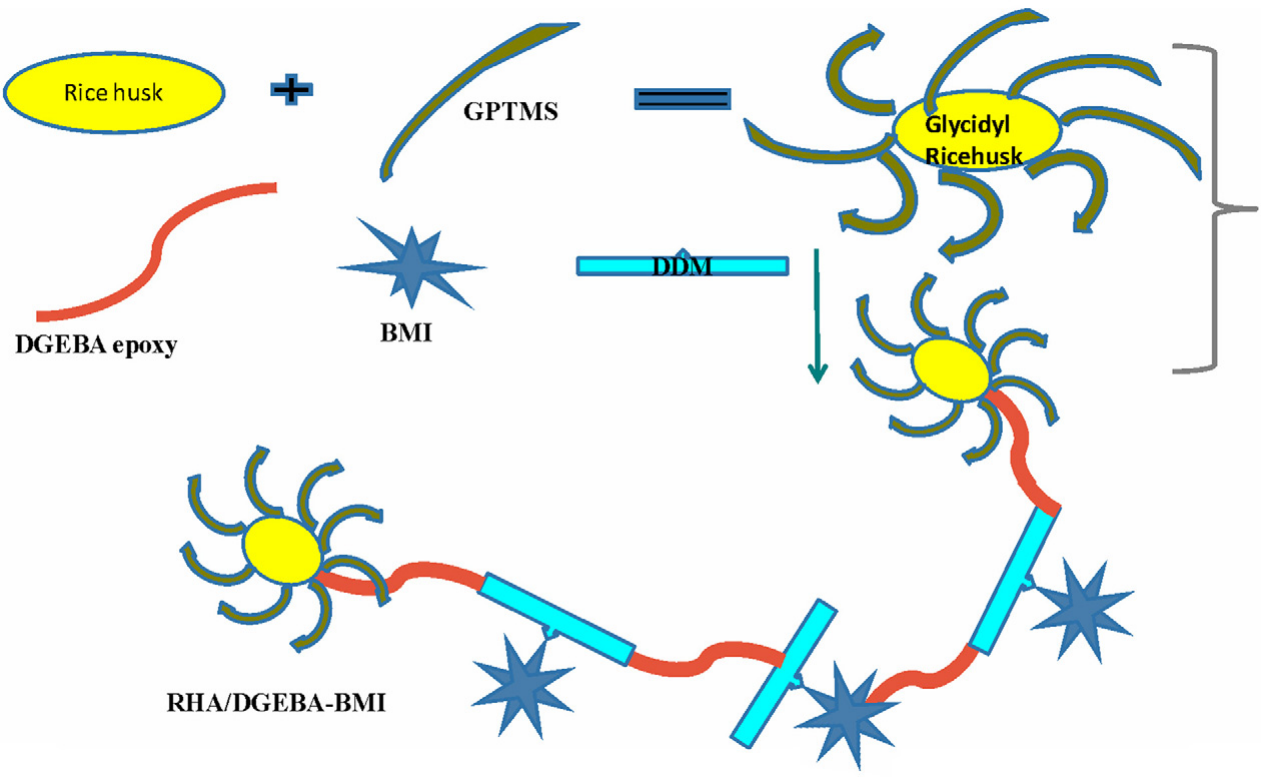
**Preparation of surface functionalized rice husk ash reinforced (GRHA-EP) nanocomposites (Graphical abstract)**.

The 5 wt.% of BMI and 95 wt.% DGEBA epoxy resin were mixed with the desired amount of glycidyl functionalized rice husk ash (0.5, 1.0, 1.5 wt.%) and mechanically stirred at 50°C for 24 h. A stoichiometric amount of DDM, corresponding to epoxy equivalents was also added. The resulting product was poured into a pre-heated mold. The mold was pre heated at 120°C for an hour, to remove the moisture and trapped air. The samples were cured successively at 120°C for 2 h, post-cured at 180°C for 3 h, and finally removed from the mold and characterized. The preparation of the RHA/DGEBA-BMI nanocomposites is illustrated in Scheme 1 and in Figure 2.

### Instrumentation

Fourier transform infrared (FT-IR) spectra for the samples were recorded on a Perkin Elmer 6X FT-IR spectrometer. The glass transition temperature (Tg) of the samples was determined, using DSC 200 PC differential scanning calorimeter (DSC) (Netzsch Gerateban GmbH). Thermogravimetric analysis (TGA) was carried out, using the DSTA 409 PC analyzer (Netzsch Gerateban GmbH). The tensile (stress–strain) properties were determined, using INSTRON (Model 6025 UK) as per ASTM D 3039. The flexural properties were measured by the INSTRON (Model 6025 UK) as per ASTM D 790. The un-notched Izod impact strength of each sample was studied as per ASTM D 256. The water absorption behavior of the samples was tested as per ASTM D 570. The percentage of water absorbed by the specimen was calculated, using the following equation:

(1) $$\%\text{Water absorption}=({\text{w}}_{2}-{\text{w}}_{1})\times 100/{\text{w}}_{1}$$

where w_1_ is the initial weight of the sample and w_2_ is the weight of the sample after immersion in distilled water for 48 h at 30°C. The dielectric studies of the neat epoxy and RHA/DGEBA-BMI nanocomposites were determined with the help of an impedance analyser. Contact angle measurements were carried out using 210 a Rame-hart Inc. goniometer (Succasunna, NJ, USA) with 5 μl of deionized water and diiodomethane (DIM).

X-ray diffraction patterns were recorded at room temperature, by monitoring the diffraction angle 2θ from 10 to 70° as the standard, on a Rich Seifert (Model 3000) X-ray powder diffractometer. The surface morphology of the fractured surface of the samples was examined, using a scanning electron microscope (SEM; JEOL JSM Model 6360). A JEOL JEM-3010 analytical transmission electron microscope, operating at 80 kV with a measured point-to-point resolution of 0.23 nm, was used to characterize the phase morphology of the developed nanocomposites. TEM samples were prepared by dissolving the powdered composite samples in ethanol mounted on carbon-coated Cu TEM grids and dried for 1 h at 70°C to form a film of <100 nm.

## Results and discussion

### FT-IR spectra of RHA/DGEBA-BMI nanocomposites

**Figure 3:**
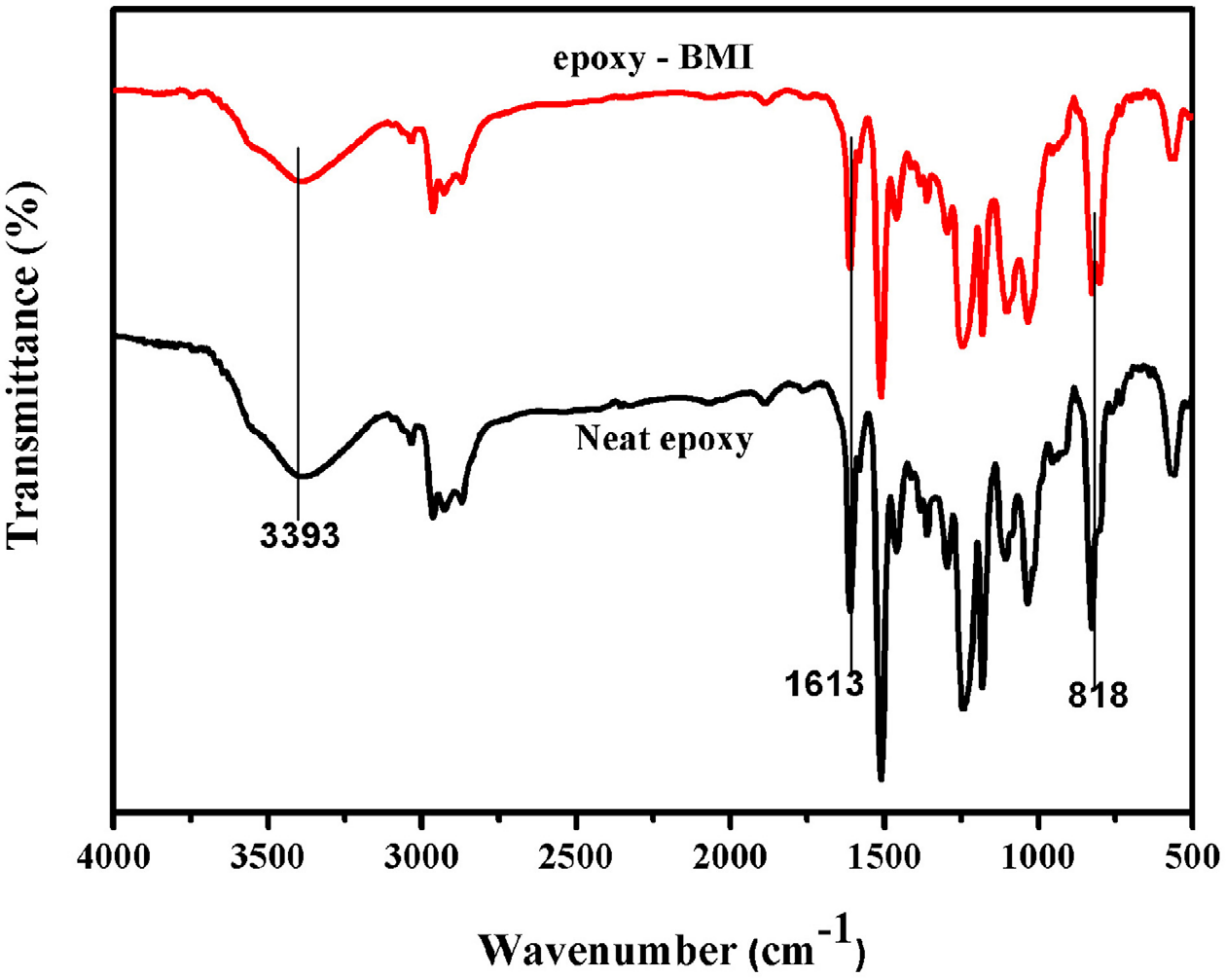
**FT-IR spectra of neat epoxy and epoxy-BMI**.

**Figure 4:**
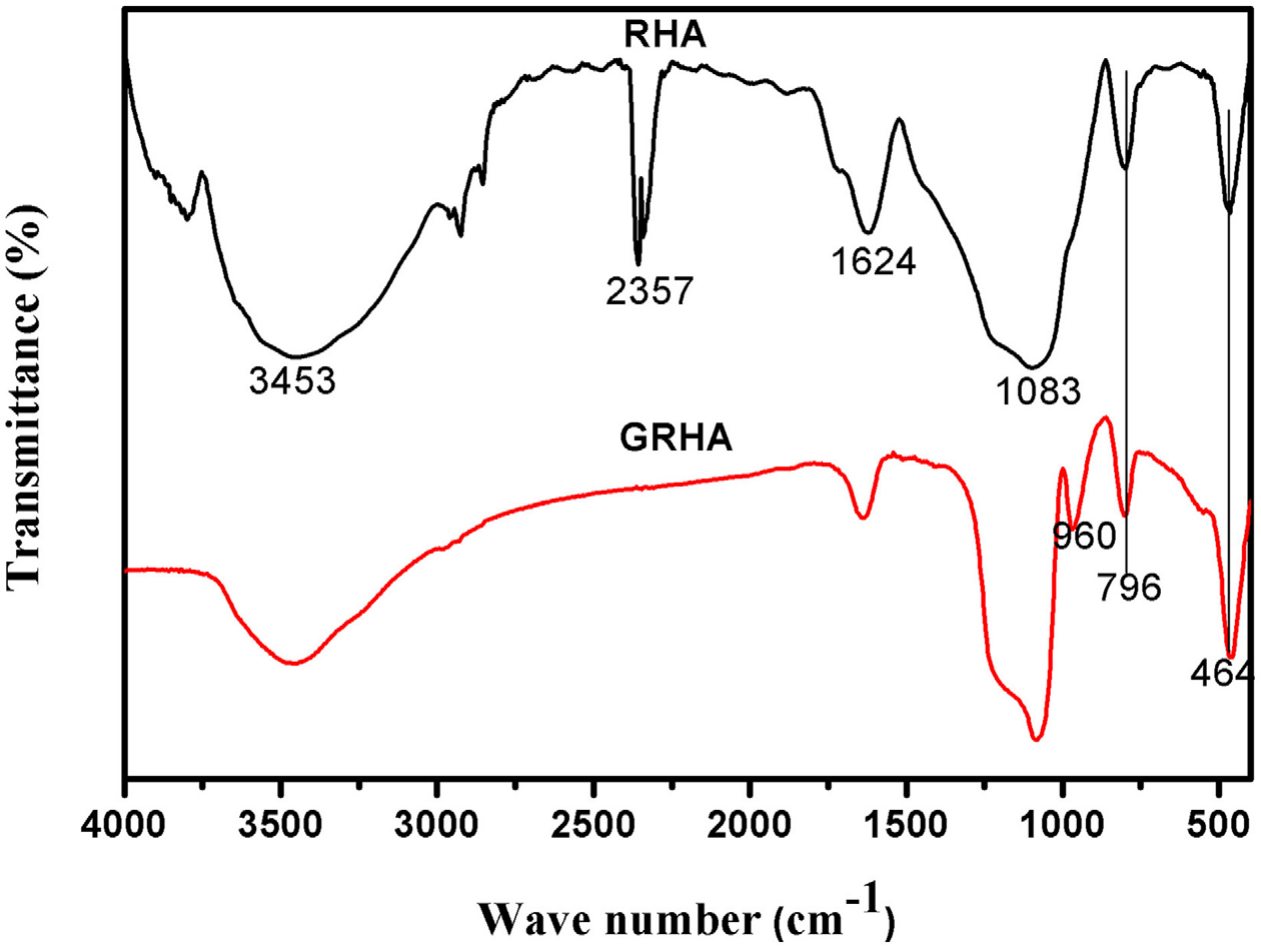
**FT-IR spectra of neat rice hush ash (RHA) and glycidyl functionalized rice husk ash**.

**Figure 5:**
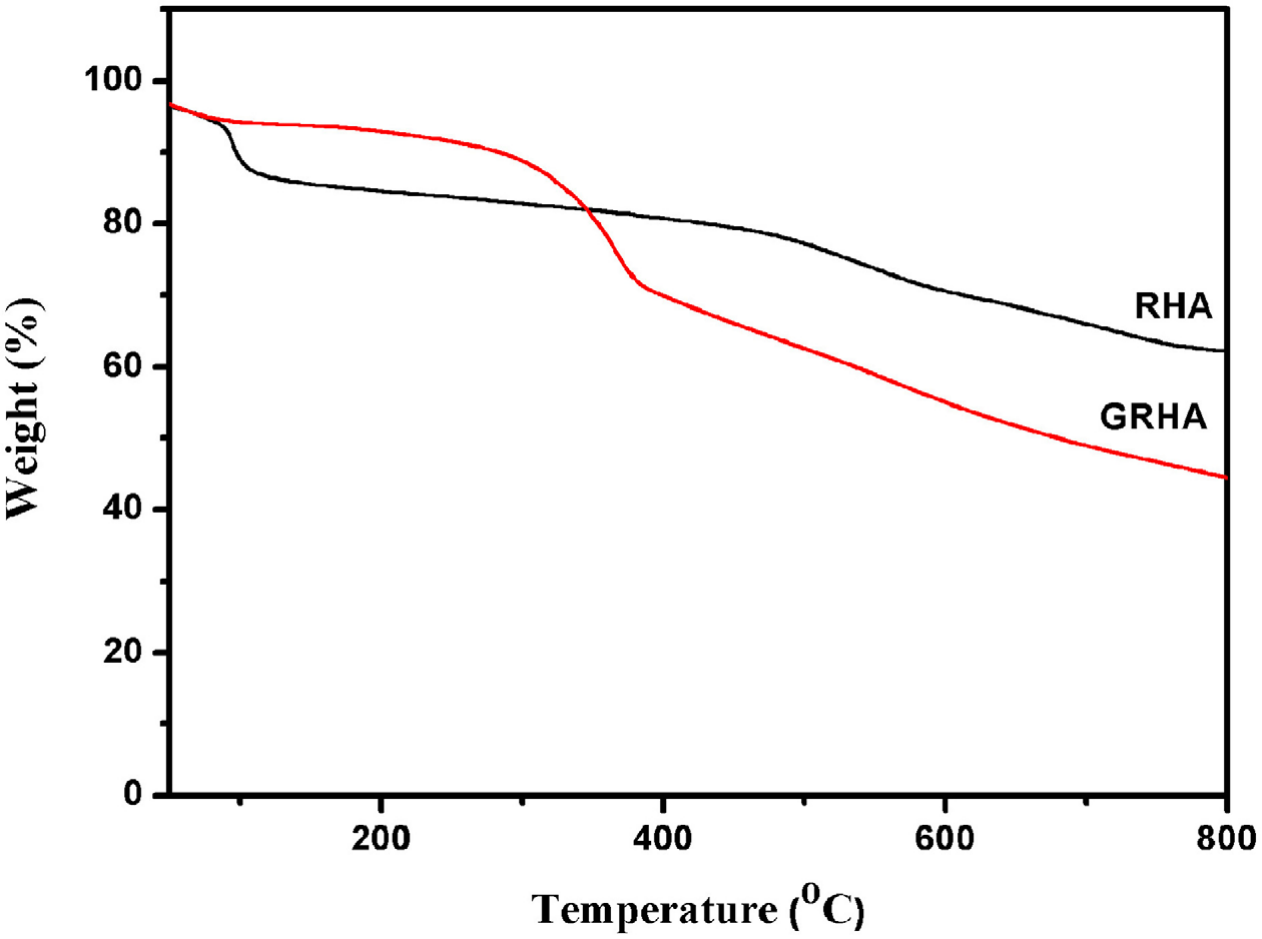
**TGA of RHA and glycidyl functionalized rice husk ash**.

Figure 3 showed the FT-IR spectra of neat epoxy and bismaleimide reinforced epoxy system, where the two set of epoxies showed their similar behavior with identical peaks. Figure 4 showed the FT-IR spectra of neat rice husk ash (RHA) and glycidyl functionalized rice husk (GRHA). The FT-IR observed for neat rice husk ash (RHA) shows the peak for OH at 3434 cm^−1^. The absence of peak at 2338 cm^−1^ is due to the chain lengthening with GPTMS and the presence of peak at 965 cm^−1^ in GRHA confirms the glycidyl functionality has been introduced into RHA. The presence of broad peak at 3468 cm^−1^ shows the stretching vibration of O-H bond. The TGA thermogram from Figure 5 also confirms the formation of glycidyl functionality in GRHA, whereas the rice husk ash possess 78 wt.% of char yield at 800°C and that of GRHA with only 65 wt.% of char yield.

**Figure 6:**
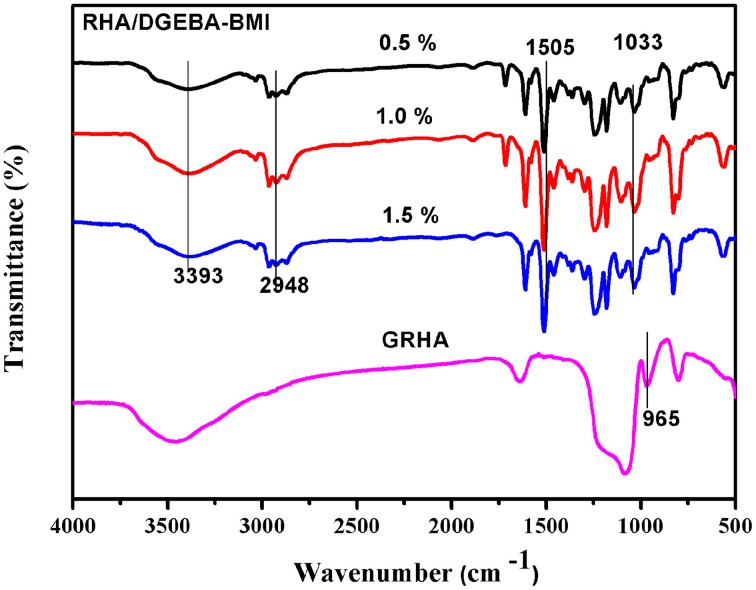
**FT-IR spectra of GRHA and RHA/DGEBA-BMI composites**.

The reaction between the glycidyl functionalized RHA, BMI and epoxy was confirmed by the FT-IR spectra and showed in Figure 6. The peak at 965 cm^−1^ in GRHA indicates the glycidyl functionality in the rice husk ash, where as its absence in the composites confirms the complete curing reaction takes place between the matrix and the reinforcements. The cleavage of oxirane ring and the formation of –OH linkage were also confirmed the occurrence of polymeric network structure. The peak at 1505 cm^−1^ indicates the -Si-O-Si- linkages by the silica group in the rice husk ash. The peaks at 1033 and 2948 cm^−1^ indicates the symmetric and asymmetric CH_2_ stretching respectively. The OH group confirms the band appearance at 3393 cm^−1^. This clearly confirms that the ring opening polymerization takes place between the epoxy, BMI, DDM and the functionalized rice husk ash during curing process.

### Effect of thermal properties of RHA/DGEBA-BMI nanocomposites

**Figure 7:**
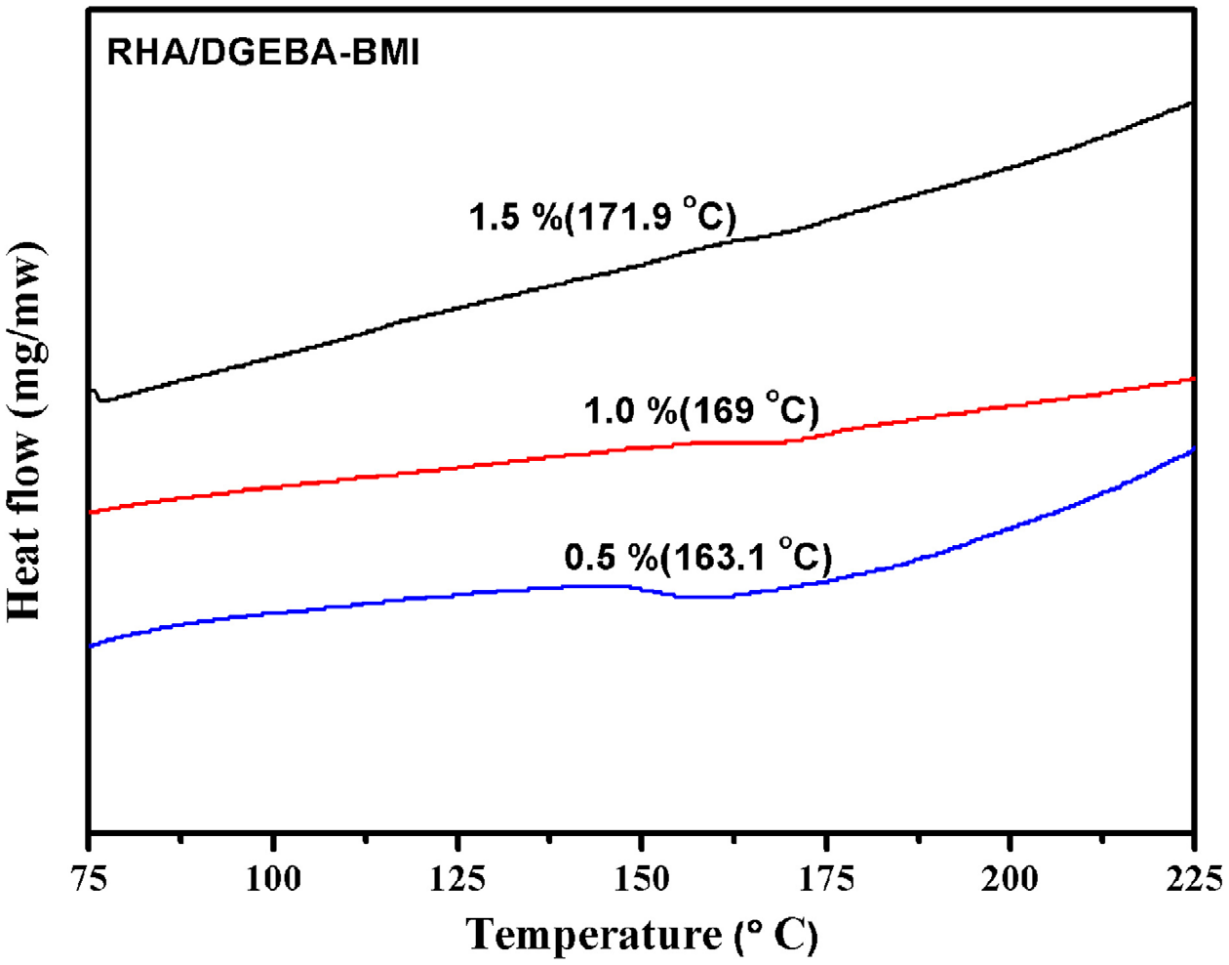
**DSC thermogram of RHA-DGEBA/BMI nanocomposites**.

The value of glass transition temperature (Tg) of the neat epoxy matrix and the RHA reinforced bismaleimide toughened epoxy composites were obtained from DSC and the data are presented in Table 1 and showed in Figure 7. In the present work RHA/DGEBA-BMI exhibited significantly higher values of Tg than that of the neat epoxy matrix. The Tg value of neat epoxy is 162°C, whereas for varying weight percentages reinforced (0.5, 1.0, and 1.5 wt.%) RHA/DGEBA-BMI nanocomposites possess 163.1, 169, and 171.9°C respectively. The higher values of Tg may be explained by the introduction of silica network by the rice husk ash and the homopolymerization of BMI through Michael addition reaction via hydrogen bonding interaction, which restricts the mobility of epoxy network. An entangled three-dimensional rigid structure was also formed due to the chemical interaction of GPTMS with the rice husk ash, BMI and epoxy (Nagendiran et al., 2008).

**Table 1:** **Thermal properties of neat epoxy and RHA/DGEBA-BMI nanocomposites**.

**Sample**	**Percentage composition**	**Tg (°C)**	**Degradation Temperature (°C)**		**Char yield at 800°C (%)**	**Water absorption (%)**	**Dielectric constant (1 MHz)**
			**40%**	**60%**			
Neat epoxy resin	100/0/0	162.0	393	420.0	0	0.123	4.50
BMI-EP	00/95/5	167.0	376.5	404.2	13.0	0.088	3.96
RHA/DGEBA-BMI	0.5/95/5	163.1	387.8	424.0	14.9	0.012	2.65
RHA/DGEBA-BMI	1.0/95/5	169.0	401.6	476.5	16.7	0.008	2.43
RHA/DGEBA-BMI	1.5/95/5	171.9	410.1	507.4	17.8	0.003	1.88

**Figure 8:**
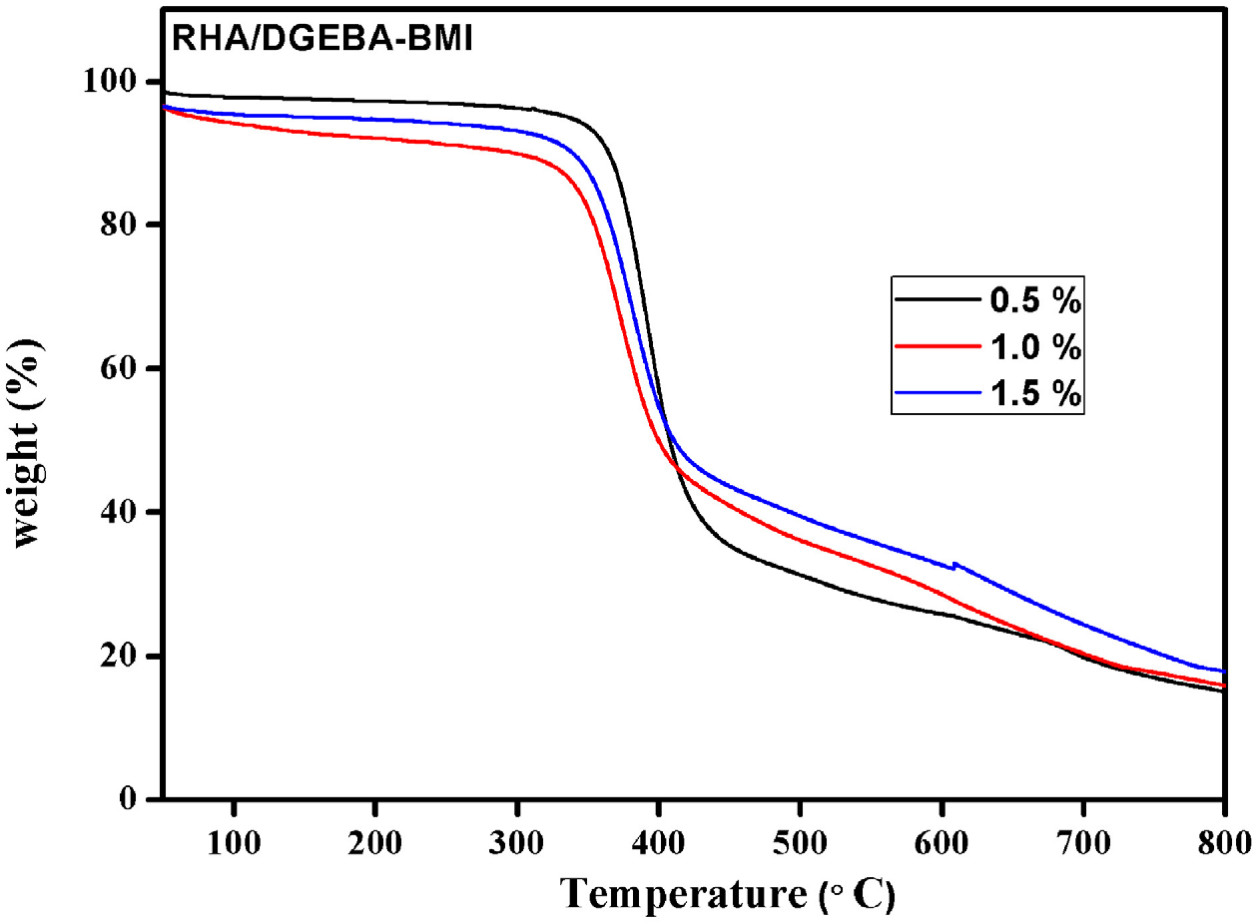
**Thermogram of RHA/DGEBA-BMI nanocomposites**.

The thermal degradation behavior of RHA/DGEBA-BMI composites are analyzed using TGA and the data are presented in Figure 8 and Table 1. The 40% weight loss for neat epoxy, BMI-EP, 0.5, 1.0, and 1.5 wt% RHA/DGEBA-BMI nanocomposites were occurred at the temperature of 393, 376.5, 387.8, 401.6, and 410.1°C respectively. Similarly it was noticed that the 60% wt. loss of the composites occurred at the temperature of 420.0, 404.2, 424.0, 476.5, and 507.5°C respectievely. The introduction of BMI into the epoxy first degrades at lower temperature whereas the surface functionalized rice husk ash reinforcement into the matrix gradually increased the thermal stability of the composites (Table 1). This may be due to the efficient interaction of the rice husk ash with BMI and the organic polymeric backbone makes the composites in a state of three dimensional cross linked network structures. As the varying weight % of glycidyl functionalized RHA and 5.0% wt. of BMI content introduced into the epoxy matrix, the system prolongs the degradation temperature of the composites due to the restriction of chain mobility and hinders the movement of molecules (Lin et al., 2007). For example, under nitrogen atmosphere, the char yield of 0.5, 1.0, and 1.5 wt.% rice husk ash reinforced bismaleimide toughened epoxy systems are 14.9, 16.7, and 17.8% respectively (Nagendiran et al., 2010) (Figure 8). The degradation of imide containing functional groups in a relatively high temperature region played an important role in enhancing flame retardation.

### Mechanical behavior of RHA/DGEBA-BMI nanocomposites

Table 2 presents the mechanical properties of neat epoxy, BMI-EP and the RHA/DGEBA-BMI nanocomposites. An incorporation of varying weight percentages (0.5, 1.0, and 1.5 wt.%) of RHA into 5.0 wt.% BMI and epoxy matrix increases the values of tensile and flexural strength. The maximum load bearing capacity of a material per unit area when it is stretched is calculated from tensile values. For neat epoxy the value of tensile strength is 61.3 MPa and for BMI-EP, 0.5, 1.0, and 1.5 wt.% reinforced nanocomposites there is an increment in the values of tensile strength to 76.8, 84.5, 99.8, and 115.9 MPa respectively. The chemically active silica present in the rice husk ash forms a strong covalent linkage with BMI and epoxy results an inter-crosslinked network structure in the reinforced composites primarily improved the value of tensile strength. The curative DDM also open its NH-OH linkage and forms an entanglement molecular structure. Flexural strength values of neat epoxy, BMI-EP and RHA/DGEBA-BMI composites are 106.0, 119.5, 125.0 147.2, and 163.1 MPa respectively. The values of flexural strength for RHA/DGEBA-BMI composites are due to the homogenous dispersion of RHA reinforcement and the BMI toughener into the epoxy matrix which supported the stress transferred from polymer matrix. It was also clearly observed from the SEM analysis that no void growth formation or crack formation occurred in the resulting reinforced composites. Moreover, the addition of rice husk ash and the BMI content into the polymer matrix enhanced the values of tensile and flexural modulus significantly. The increase in modulus value is showing the stiffness of RHA/DGEBA-BMI reinforced composites. This improvement is attributed to the relatively lower strain rates of the composites. An observation similar to this was also noticed in the case of hardness behavior. Due to the increment of the loading, the values of hardness enhanced from 84 to 214.5. Further it was also observed that the reinforced composites have resistant to penetration, scratching and indentation of other foreign agents (Zulfiqar et al., 2007).

**Table 2:** **Mechanical properties of neat epoxy and RHA/DGEBA-BMI nanocomposites**.

**Sample**	**Percentage composition**	**Tensile strength (MPa)**	**Tensile modulus (GPa)**	**Flexural strength (MPa)**	**Flexural modulus (GPa)**	**Impact strength (Jm^−1^)**	**Hardness HV1.2**
Neat epoxy	100/0/0	61.3	2.71	106.0	2.37	101.7	84
BMI-EP	00/95/5	76.8	3.57	119.5	2.69	110.0	121.6
RHA/DGEBA-BMI	0.5/95/5	84.5	3.84	125.0	2.78	113.1	130.0
RHA/DGEBA-BMI	1.0/95/5	99.8	4.23	147.2	3.26	119.6	166.7
RHA/DGEBA-BMI	1.5/95/5	115.9	4.78	163.1	3.54	125.8	214.5

The amount of energy absorbed per unit area is calculated by the impact strength. The values of impact strength for neat epoxy, BMI-EP, RHA/DGEBA-BMI nanocomposites are presented in Table 2. The reinforcement RHA, toughener BMI into the epoxy matrix improved the impact behavior according to their percentage content. The oxirane ring in the DGEBA molecule and –OH surface of the reinforcement induces to form hydrogen bond with the maleimide linkage which tightly holds one another and reason out for the increased values of impact strength. The primary long-chain rigid structure and possible secondary hydrogen bond formation is expected to form a chain-entangled network structure (Chanda and Rahabi, 1997). The values of impact strength of toughened and reinforced composites enhanced to a significant extent when compared to that of neat epoxy due to the flexibility and plasticizing effect imparted by the silica moiety in the rice husk ash (Hameed et al., 2007).

### Influence of water absorption dielectric behavior and contact angle of RHA/DGEBA-BMI nanocomposites

The water absorption behavior and dielectric constant of RHA/DGEBA-BMI nanocomposites was studied and presented in Table 2. The experiments showed volume change without any clustering or microvoiding which clearly confirms the water repellent character of the nanocomposites. All the surface-modified rice husk ash reinforced BMI toughened epoxy resin systems showed good resistance to moisture absorption was purely due to the rigid aromatic hydrophobic structure. The decrease in percentage water uptake of the RHA-BMI incorporated systems was due to the inherent hydrophobic nature of the RHA networks. Rice husk ash possesses its original nature that it does not absorbed water and it is also biologically non-degradable in nature. Neat epoxy matrix possesses the values of dielectric constant of 4.50 at 1 MHz whereas that of BMI-EP and 0.5, 1.0, and 1.5 wt.%. RHA/DGEBA-BMI composites are 3.96, 2.65, 2.43, and 1.88 respectively. Decreasing trend of dielectric constant indicates that the increasing incorporation of RHA and BMI which in turn reduce the dipole–dipole interaction. This behavior reduces the polarizability and increases the free volume of the resulting hybrid nanocomposites which obviously reduced the values of dielectric constant. Low water absorption and dielectric constant are very important property for a material used for insulation applications, especially those requiring stable high performance (Devaraju et al., 2013).

**Figure 9:**
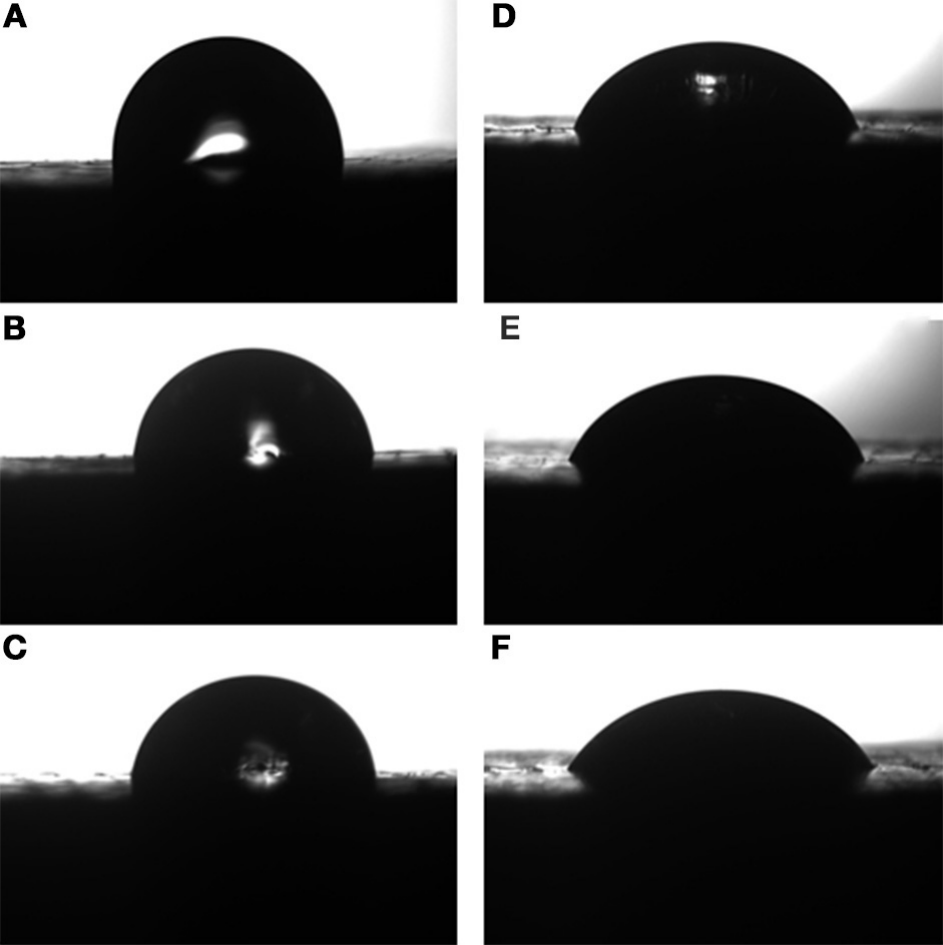
**Contact angle of (A-0.5%, B-1.0%, C-1.5% RHA-DGEBA/BMI) for diiodomethane and water (D-0.5%, E-1.0%, F-1.5% RHA-DGEBA/BMI)**.

The variation of contact angle against blend composition is given in Figure 9 and in Table 3. It can be observed that the reinforced composites possess higher values of contact angle when compared to that of neat epoxy matrix. The values of contact angle of RHA/DGEBA-BMI composites are increased from 82.7 to 89.3 and from 52.7 to 60.4 for water and DIM respectively. The lower affinity of the developed composites indicates the improvement in the hydrophobic behavior contributed by the reinforcement and toughening agent to organic matrix. This may be due to the non-polar nature of the toughened rice husk ash and the BMI which may ultimately increases the roughness of the polymer surface. The acid treatment and the glycidyl functionalization of the rice husk ash are important modification that disrupts the molecular motion of the polymer and induced the crosslinked network structure, thereby increasing the surface roughness. The roughness morphology of polymer surface was also supported by the SEM micrograph (Figure 8). The additional free energy at the surface of the composites is known as surface free energy. High-energy absorption received by the activated curing agents of –NH bonds strike the DGEBA epoxy which interacted with the toughened BMI component and to the surface of rice husk reinforcement thereby forming a network structured composites. Consequently, the developed composites reduced the surface energy and enhanced the substrate surface roughness and make the material surface become super hydrophobic (Yue et al., 2010).

**Table 3:** **Contact angle and surface energy of RHA/DGEBA-BMI nanocomposites**.

**Properties**	**Contact angle**		**Surface energy**		
	**Water**	**Diiodomethane**	**γ^d^**	**γ^p^**	**Γ**
0.5% RHA/DGEBA-BMI	82.7	52.7	13.3	10.9	46.8
1.0% RHA/DGEBA-BMI	88.7	57.8	28.3	5.1	33.5
1.5% RHA/DGEBA-BMI	89.3	60.4	27.8	19.1	24.2

### Morphological features of RHA/DGEBA-BMI nanocomposites

**Figure 10:**
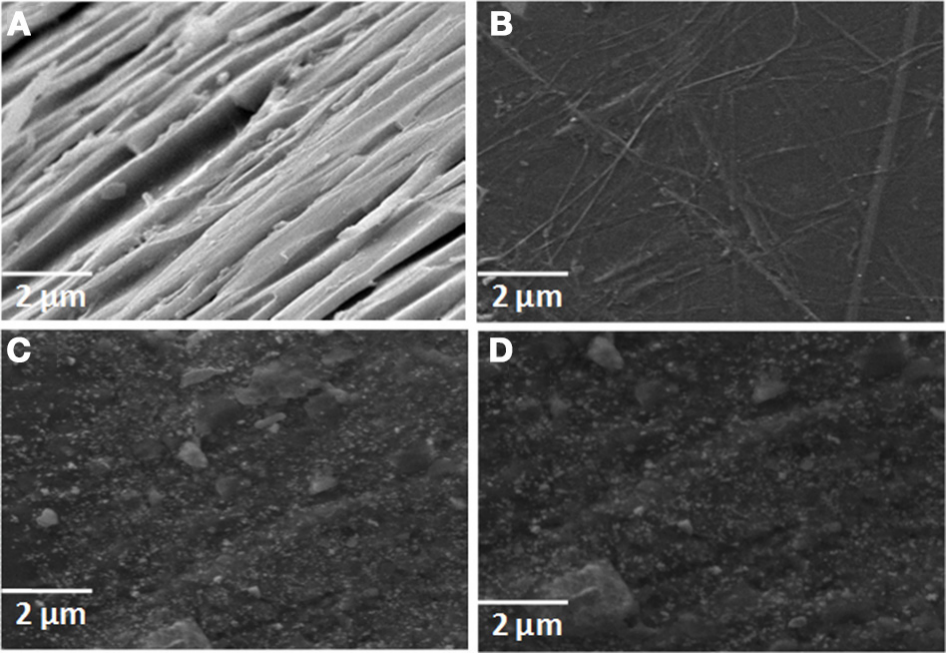
**SEM micrograph of (A) neat epoxy, (B) 0.5, (C) 1.0, (D) 1.5 wt.% RHA-DGEBA/BMI nanocomposites**.

The morphology of neat epoxy matrix and RHA/DGEBA-BMI composites were analyzed using SEM micrograph and presented in Figure 10. The neat epoxy matrix (Figure 10A) shows a smooth glassy fractured surface in different places. The brittle behavior was overcome by the addition of glycidyl functionalized rice husk ash and the BMI as toughening agent. There is no observance of crack formation or pulled out flaws in the SEM image (Figures 10B–D), which clearly provides an idea about the exfoliation of reinforcement combined with toughening agent into the matrix. At higher weight percentages also no chance for agglomeration of particles observed in the epoxy matrix which confirms the homogenous and molecular level dispersion of rice husk ash into epoxy matrix. This contributes to the improvement of thermal and mechanical properties (Devaraju et al., 2011).

**Figure 11:**
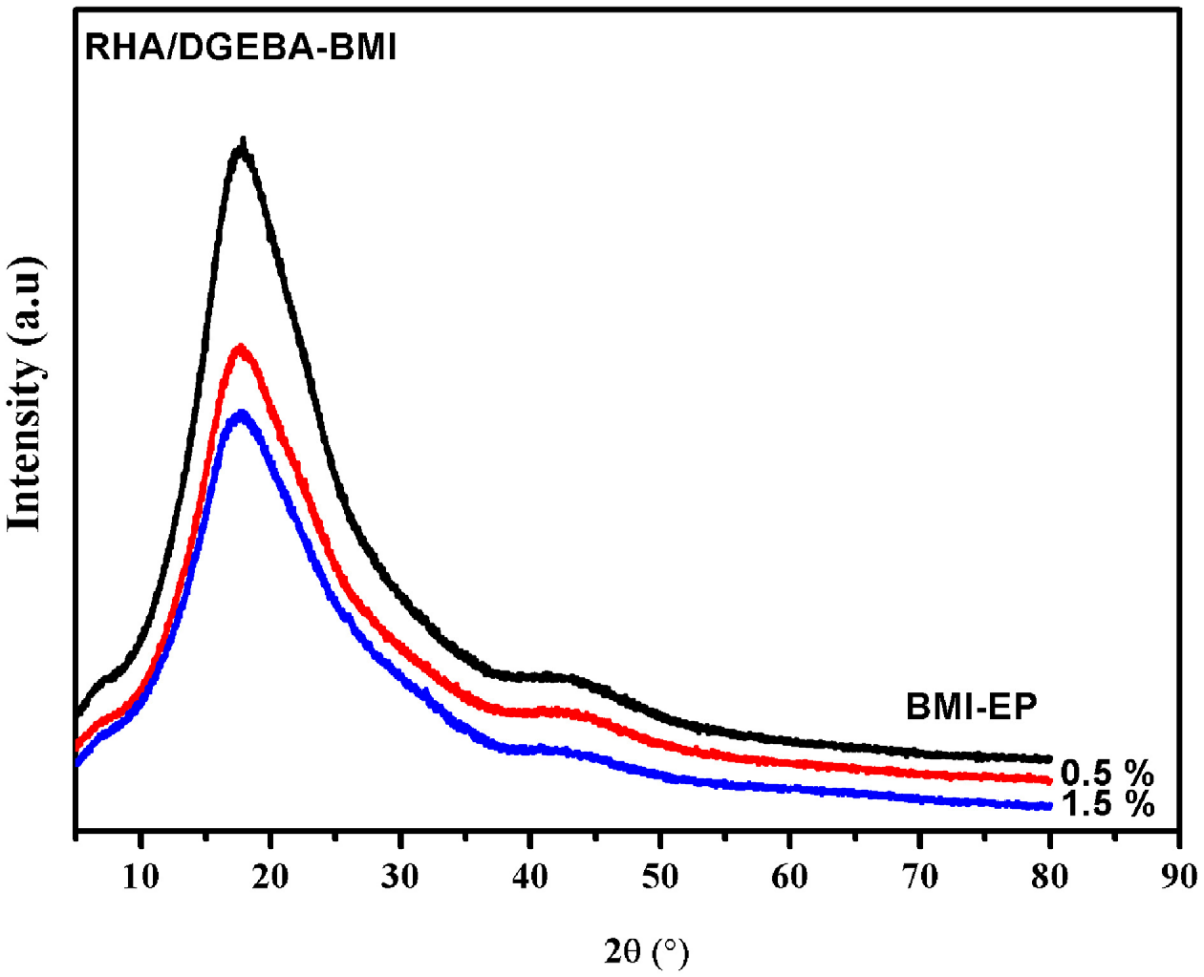
**XRD of BMI-EP, 0.5 and 1.5 wt.% RHA/DGEBA-BMI nanocomposites**.

XRD analysis is used to investigate the 0.5 and 1.5% RHA/DGEBA-BMI composites and the diffractograms of the samples are presented in Figure 11. From Figure 11, it is evident that there is no diffraction peak observed for epoxy composites. The absence of the diffraction peak in the case of reinforced composites is due to the complete exfoliation of the RHA-BMI into the epoxy network structure. The observance of peak at 2θ = 20° showed the amorphous nature and the uniform level dispersion of RHA-BMI concurrently confirms the efficient and effective compatibility between the RHA-BMI and the epoxy matrix. The RHA-BMI dispersion is homogenously observed in the form of individual layers within the polymer matrix and leads to form exfoliated composites which attributes to the improvement of properties of the resulting nanocomposites.

**Figure 12:**
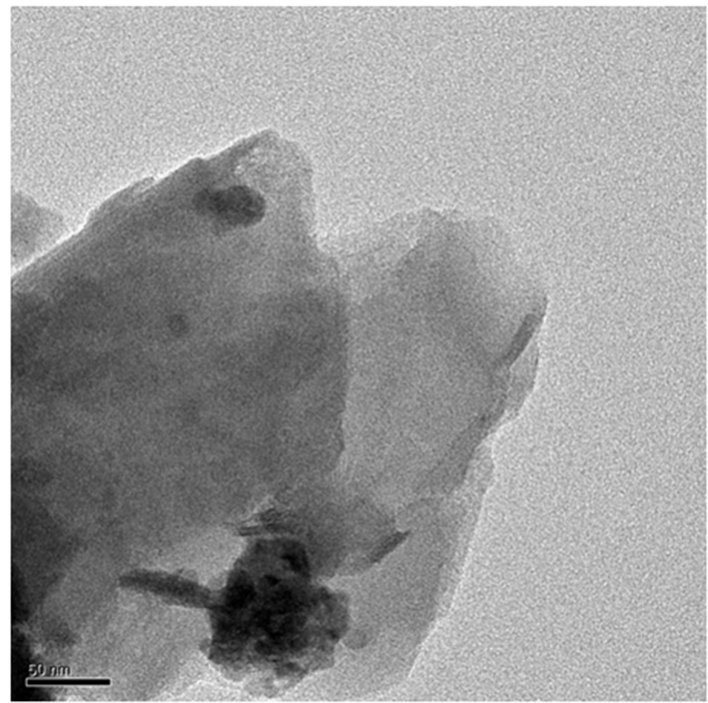
**TEM micrograph 1.0% RHA-DGEBA/BMI nanocomposites**.

The TEM micrograph for 1.0 wt.% RHA/DGEBA-BMI nanocomposites is shown in Figure 12. The nanozized distribution of RHA and BMI content can be viewed from the TEM micrograph where the smaller particles correspond to RHA and the bigger particles related to BMI. It was also ascertained that the dark portion of the pictures are due to the homogenous dispersion of rice husk ash and BMI into the epoxy matrix without any crack. No accumulation mode was observed in the particle number size distribution, the particles on the left top corner were clearly visible which indicates the presence of rice husk ash reinforcement and bottom corner represents BMI respectively.

**Figure 13:**
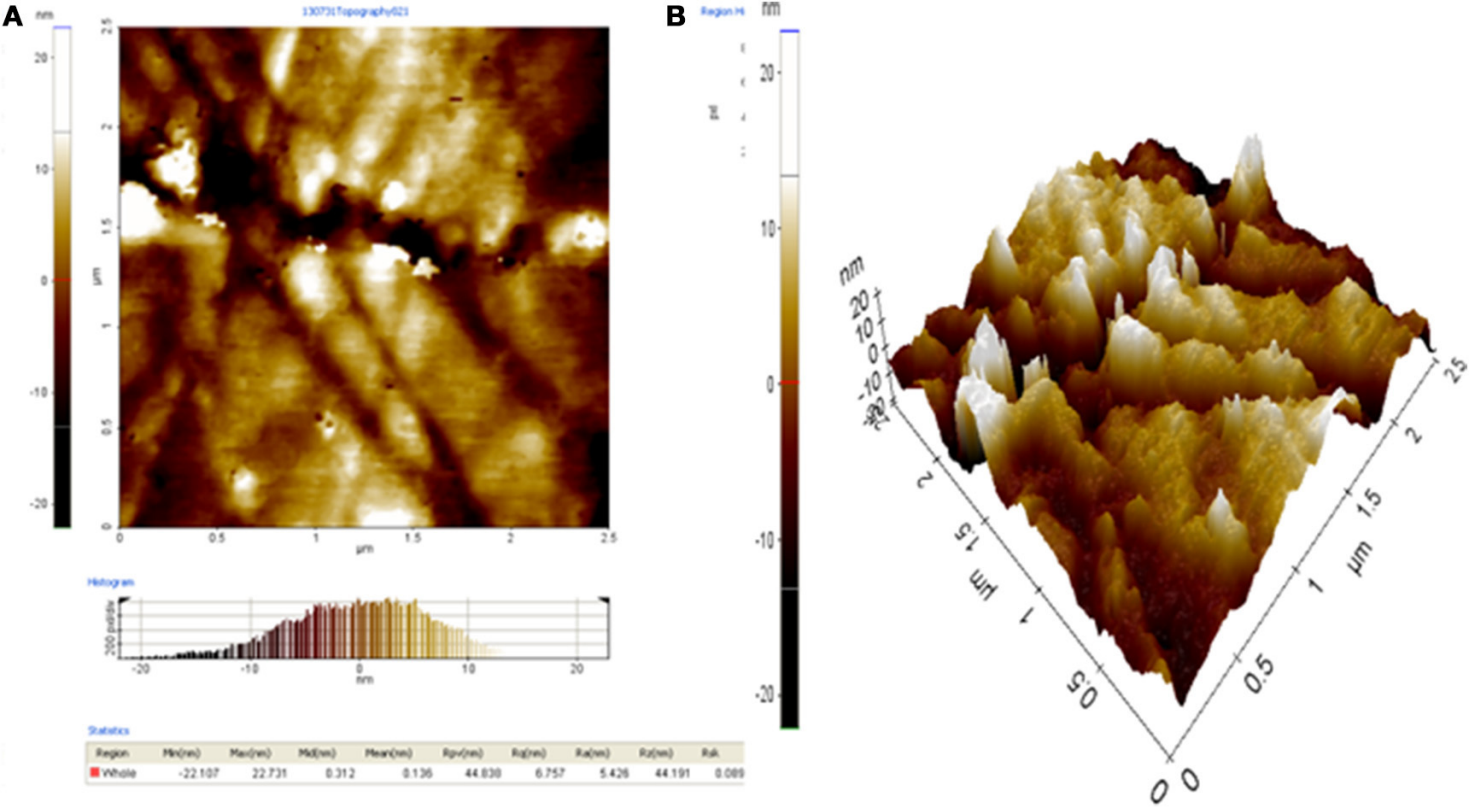
**AFM microphotographs of 1.0% RHA-DGEGBA/BMI nanocomposites (A) 2D image (B) 3D image**..

The surface topology of 1.0% wt. RHA/DGEBA-BMI in 2-D and 3-D was characterized using the AFM technique and are shown in Figure 13. The surface of the hybrid thin film is quite smooth and has few visible defects, indicating that the developed composites have a molecular level dispersion of organic and inorganic networks, silica particles and BMI in RHA/DGEBA-BMI are distributed uniformly in the organic phase. The phase images also indicate that the BMI and reinforcement domains were uniformly dispersed throughout the matrix. This is further supported by SEM analysis that the hybrid exhibits excellent structural uniformity, with no cracks or flaws. The surface morphology of the hybrid materials is of great importance for industrial applications. The compatability between the epoxy, BMI and inorganic rice husk ash had a great significant in thermal and mechanical properties.

## Conclusion

Varying weight percentages of surface functionalized rice husk ash reinforced BMI toughened epoxy nanocomposites (0.5, 1.0, and 1.5 wt.% RHA/DGEBA-BMI) were developed and their mechanical, thermal, dielectric and surface morphology were studied. The developed composites possess improved thermo-mechanical properties due to excellent covalent interaction between them. RHA/DGEBA-BMI exhibited with higher values of glass transition, delay in degradation of the composites, higher char yield and excellent flame retardant character showed that the composite materials can withstand at higher temperature than that of neat epoxy matrix. The homogenous level of dispersion and exfoliation of the composites were confirmed by SEM, TEM, AFM, and XRD analysis. The hydrophobic nature of the composite samples is well understood from the increasing value of contact angle and the surface roughness of the composites experienced by the RHA and the BMI. The enhanced properties of the developed composites make it to use in the form of coatings, adhesives and matrices which can be utilized for the fabrication of advanced composite components in the place of conventional epoxy for high performance applications.

### Conflict of interest statement

The authors declare that the research was conducted in the absence of any commercial or financial relationships that could be construed as a potential conflict of interest.
